# Concentration-Emission Matrix (CEM) Spectroscopy Combined with GA-SVM: An Analytical Method to Recognize Oil Species in Marine

**DOI:** 10.3390/molecules25215124

**Published:** 2020-11-04

**Authors:** Yunan Chen, Ruifang Yang, Nanjing Zhao, Wei Zhu, Xiaowei Chen, Ruiqi Zhang, Jianguo Liu, Wenqing Liu

**Affiliations:** 1 Key Laboratory of Environmental Optics and Technology, Anhui Institute of Optics and Fine Mechanics, Chinese Academy of Sciences, Hefei 230031, China; ynchen@aiofm.ac.cn (Y.C.); rfyang@aiofm.ac.cn (R.Y.); wzhu@aiofm.ac.cn (W.Z.); xwchen@aiofm.ac.cn (X.C.); rqzhang@aiofm.ac.cn (R.Z.); jgliu@aiofm.ac.cn (J.L.); wqliu@aiofm.ac.cn (W.L.); 2 Hefei Institutes of Physical Science, University of Science and Technology of China, Hefei 230026, China; 3 Key Laboratory of Optical Monitoring Technology for Environment, Anhui Province, Hefei 230031, China

**Keywords:** concentration-emission matrix, PCA, genic algorithm, SVM, oil species recognition

## Abstract

The establishment and development of a set of methods of oil accurate recognition in a different environment are of great significance to the effective management of oil spill pollution. In this work, the concentration-emission matrix (CEM) is formed by introducing the concentration dimension. The principal component analysis (PCA) is applied to extract the spectral feature. The classification methods, such as Probabilistic Neural Networks (PNNs) and Genic Algorithm optimization Support Vector Machine (SVM) parameters (GA-SVM), are used for oil identification and the recognition accuracies of the two classification methods are compared. The results show that the GA-SVM combined with PCA has the highest recognition accuracy for different oils. The proposed approach has great potential in rapid and accurate oil source identification.

## 1. Introduction

The types and ratios of polycyclic aromatic hydrocarbons (PAHs) and their derivatives are significantly different in different kinds of spill oils, which provides a theoretical basis for oil identification. Traditional methods such as gas chromatography-mass spectroscopy (GC-MS) [1,2,3,4], gas chromatography-flame ionization detection (GC-FID) [5,6,7] and high-performance liquid chromatography (HPLC) [8,9,10] have a high resolution; they are considered reliable analytical methods. However, they have complicated pretreatment procedures and require a large amount of solvents. In addition, traditional analytical instruments can only be operated in a laboratory environment, which is challenging to meet the on-site analysis and quickly give the identification results in the case of oil spill emergency treatment [11,12,13,14]. 

The fluorescence technique is a sensitive, rapid and non-destructive screening method that can be used as a complement to traditional methods [15]. The oil-bearing samples contain a variety of polycyclic aromatic hydrocarbons, which are stimulated to produce fluorescence. So, the fluorescent nature of the petroleum product is related to the electronic structure of its aromatic compounds. Therefore, some researchers have conducted study of the fluorescence properties of petroleum products to establish reliable fluorescence analysis methods for oil spills [16,17,18,19,20]. Mirnaghi et al. used the EEM fluorescence spectroscopy, PARAFAC method and principal component analysis (PCA) to identify and quantify unknown spilled oil, thereby delivering a preliminary evaluation of the petroleum products as soon as possible [21].

However, one major problem in the identification of oil samples by the fluorescence technique is that the shapes of fluorescence spectra are sensitive to the concentration, which has been reported by some researchers [22,23,24]. At a higher concentration, the fluorescence of the low-ring PAHs would be quenched, and the fluorescence information of the high-ring PAHs would be retained. Spectral changes caused by red shift behavior can affect the oil species recognition results based on fluorescence spectra technique [25,26,27,28]. Therefore, the PAH_S_ composition ratio of oils cannot be adequately represented by only one certain concentration. 

The other problem is how to choose the appropriate feature extraction algorithm and recognition algorithm to identify the species of oils. Recently, Machine learning has been widely used to solve pattern recognition, data mining, and other problems, such as Convolutional Neural Networks (CNN) [29] and Probabilistic Neural Networks (PNNs) [30]. However, these neural network algorithms are not suitable for the cases with small sample datasets. Support Vector Machine (SVM) is state-of-the-art data processing that has unique advantages in dealing with complex problems such as finite samples and high-dimensional nonlinear data [31,32,33,34,35]. Rios-Reina et al. distinguished the wine vinegar in the Spanish Designation of Protected Areas based on support vector machine, which obtained the better classification results (>92% classification rate) [36]. The performance of the SVM is closely related to its penalty factors and kernel parameters. Therefore, choosing the appropriate parameters is the key to improve classification accuracy. Currently, there are many parameter optimization methods. Chang et al. used particle swarm optimization (PSO) algorithm to optimize SVM parameters, which improved the classification effect of image textures [37]. Zhang et al. used the ant colony algorithm to optimize SVM parameters and improved the classification performance of SVM [38]. Li et al. applied the cross validation (CV) and genetic algorithm (GA) to optimize the parameters of SVM, respectively, and the results showed that GA-SVM can perform rapid and high accuracy recognition [39]. Nevertheless, there are few reports about the identification of fluorescence spectra based on SVM. 

In order to solve the above problems, the three-dimension concentration-emission matrix (CEM) spectra are formed by introducing the concentration dimension. It not only includes the wavelength and intensity of the fluorescence peak, but also includes the variation of the fluorescence spectra with the concentrations, which can express all the fluorescence information from low-ring PAHs to high-ring PHAs. For better extraction the spectral information, PCA is used to extract feature of the CEM spectra. The GA-SVM algorithm and PNNs are used to recognize the six kinds of oils in different environmental condition, the recognition accuracy of the two algorithms are compared. Our research provides a method for fast and accurate identification of oil samples.

## 2. Results and Discussion

### 2.1. The CEM Characteristics of Different Oil Samples

**Figure 1:**
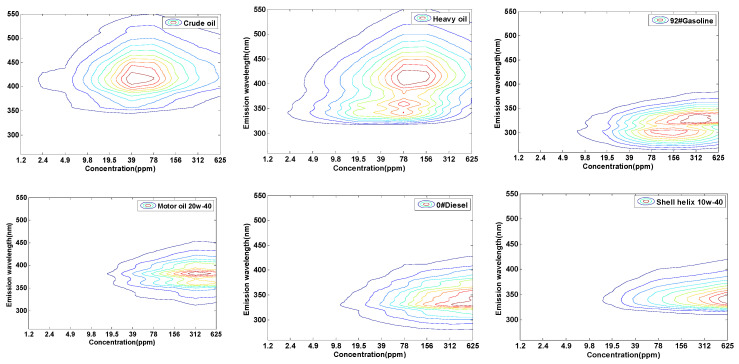
Concentration-emission matrix (CEM) spectra of 6 kinds of oil samples.

The three-dimensional concentration-emission matrix (CEM) has been formed by obtaining the fluorescence emission spectra at the excitation wavelength of 266 nm with 10 series of concentrations. The emission spectrum of each concentration of oil is a matrix with 146 rows and 1 column, so the CEM composed of 10 concentrations of emission spectra is a matrix with 146 rows and 10 columns. Figure 1 shows the CEM spectra of six kinds of oils. 

The CEM spectra of crude oil have one fluorescence peaks located at $x/\lambda_{em}$ of 39 ppm/420 nm. While heavy oil has the two fluorescence areas with the center at 78 ppm/350 nm and 78 ppm/420 nm. 0#diesel and shell helix 10w-40 have similar spectral characteristics, which both have one peak of CEM spectra with the centers at $\;x/\lambda_{em}$ 625 ppm/340 nm. The 92#gasoline and motor oil 20w-40 both have two peaks, the components ($x/\lambda_{em}\;$= 156 ppm/300 nm and $\;x/\lambda_{em}$= 312 ppm/340 nm) refer to 92#gasoline and the components ($x/\lambda_{em}$= 312 ppm/360 nm and $x/\lambda_{em}$= 312 ppm/390 nm) refer to motor oil 20w-40. As the concentration of the oil sample increases, the fluorescence spectra would produce red-shift behavior. Different kinds of oils have different components, so their fluorescence red shift behavior differs vastly. Thus, this provides the basis for the identification of different types of oils using CEM spectroscopy. 

The fluorescence characteristics of the water environment in the different areas could be different and the fluorescence spectra of the oils could change during the weathering process, which would interfere with the fluorescence spectra of oils. In this work, the weathering processes and the different seawater are taken into consideration to establish a spectral database for oil recognition. Five oil stock solutions with different weathering times (0, 10, 20, 30 and 40 days) are prepared for each oil through weathering experiments, thereby 30 stock solutions are prepared. Water samples from six different locations (five different areas in Yellow sea-Bohai sea and one Dongpu Reservoir water) are collected to prepare the solution. Six kinds of water samples are added to 30 weathering stock solutions to prepare a series of working solutions. The fluorescence spectra of different concentrations of samples are obtained, and the CEM spectra of different kinds of oils are formed, with a total of 180 CEM spectra. 

### 2.2. Features Extraction of Oil Samples Based on PCA

**Figure 2:**
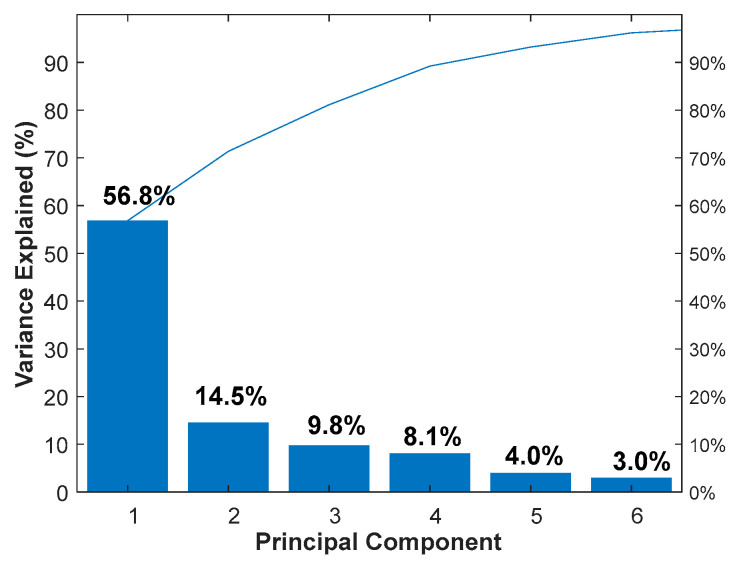
Contribution rates of the first six principal components.

Feature extraction is essential to the performance of oil spectra recognition system. PCA is usually used for feature extraction and dimensionality reduction. PCA is generally used to process the first-order vector of each sample (such as the emission spectra). For the second-order matrix (such as EEM or CEM), it is necessary to deform the data to meet the processing requirements. In this work, the CEM spectrum is a (146 × 10) matrix, which is transformed into a row-vector (1 × 1460) by unfolding the (146 × 10) matrix end to end. The 180 CEM spectra are expanded into vectors to form a 180 × 1460 matrix. Then, the dataset is standardized to a unit scale (mean = 0 and variance = 1) based on the mean and standard deviation of the original data. The spectral features is extracted using PCA, and the variance explained is shown in Figure 2. It can be seen that the variance contribution (PC1: 56.8%; PC2: 14.5%, PC3: 9.8%; PC4: 8.1%; PC5: 4.0%; PC6: 3.0%) of the first six PCs can reach 96%. The first six principal component matrices (180 × 6) obtained by the PCA are used as feature spectra and input into different classifiers in the next stage in our algorithm design.

### 2.3. Spectra Classification Results 

The feature information of 180 × 1460 matrix is extracted by PCA to obtain the principal component matrix with 180 rows and 6 columns. The crude oil, 0#diesel, heavy oil, motor oil 20w-40, 92#gasoline, Shell helix 10w-40 are set to 1st label, 2nd label, 3rd label, 4th label, 5th label, 6th label, respectively. Each type of oil contains 30 samples, of which 20 samples are used as the training set, and the remaining 10 samples are used as the testing set. Thus, the training set contains 120 samples and the testing set contains 60 samples. 

The main parameters for GA-SVM and PNNs are shown in Table 1. The five cross-validation experiments are carried out to improve the stability of the GA-SVM model. The average of the classification accuracy is used as the performance indicator of the classifier. The experimental platform is MATLAB R2019a and the LIBSVM software package designed by Professor Lin at National Taiwan University is carried out. 

**Figure 3:**
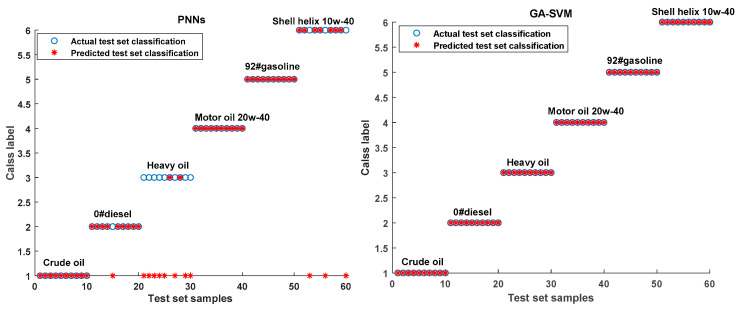
Results of actual classification and predicted classification of PNNs and GA-SVM.

As shown in Figure 3 and Table 2, the average classification rate of PNNs for six kinds of oils is 80% [48/60]. Among them, the classification accuracy of heavy oil is the lowest at 20% [2/10], and the classification accuracy of crude oil is the highest at 100% [10/10]. However, the classification accuracy of GA-SVM for each oil sample is 100%, which indicates that the prediction results of GA-SVM is significantly better than that of PNNs in spectral recognition. The poor prediction results of the PNNs may be due to its information processing paradigm, which makes it easy to fall into a local minimum. Whereas SVM is a classification method based on the principle of structural risk minimization, which can effectively avoid falling into the local optimum. It has a unique advantage in dealing with limited samples and high-dimensional nonlinear data set. In addition, the GA is chosen to optimize the kernel parameters and penalty factors of SVM, which further improve the accuracy of classification.

## 3. Materials and Methods

### 3.1. Samples Preparation

Six different types of oils: 0#diesel, crude oil, heavy oil, 92#gasoline, shell helix 10w-40, and motor oil 20w-40. The oil sample was weighted by electronic balance and dissolved in isopropanol, which was placed in an ultrasonic oscillator to be fully dissolved. After waiting for 30 min, the supernatant was taken as the stock solution with a concentration of 5000 ppm of the oil sample. The concentrations of working solutions with a serial of concentrations (1.2, 2.4, 4.9, 9.8, 19.5, 39, 78, 156, 312, 625 ppm, respectively) were prepared by diluting the stock solutions.

### 3.2. Weathering Experiments 

During the 40 days weathering experiment period, the lowest temperature was 19 °C and the highest temperature was 31 °C. The weather was mainly sunny and only 9 days were rainy. The oil samples were subjected to weathering by placing 5-cm-thick oil film in a beaker placed in natural conditions for 40 days. Stock solutions (5000 ppm) were prepared by dissolving appropriate weathering samples for 10 days, 20 days, 30 days and 40 days, respectively. Due to the volatility of 92#gasoline, four stock solutions of weathering sample were prepared in 12, 24, 36 and 48 h. 

### 3.3. Water Samples

**Figure 4:**
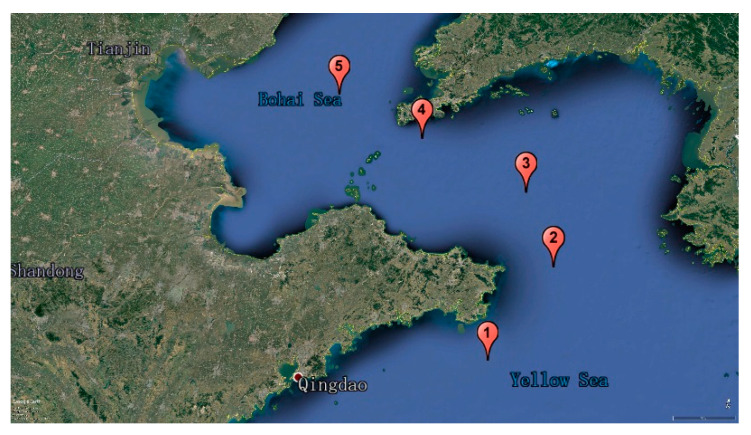
Map of seawater samples in the Bohai Sea and the Yellow Sea area.

Taking the scientific research ship “Haijian NO.101” as a platform, different seawater samples were taken at five different points in the Bohai Sea and the Yellow Sea area, as shown in Figure 4. The coordinates of the five points are as follows: 1(122.45/36.29), 2(123.18/37.28), 3(122.52/38.11), 4(121.27/38.36), 5(120.17/38.57). The reservoir water of Dongpu Reservoir in Hefei City, Anhui Province was also collected as one of the water environmental backgrounds.

### 3.4. Analytical Technique

Fluorescence measurement was performed on a Hitachi F-7000 spectrofluorometer (Hitachi, Japan). The excitation-emission matrices for three-way fluorescence spectra of each sample were record with the excitation wavelengths EX in range of 250–450 nm at a 2 nm interval and emission wavelength EM 260–550 nm at a 2 nm interval. EEMSCAT was used to remove Raman scattering and Raleigh scattering in the MATLAB environment. 

### 3.5. Principal Components Analysis (PCA)

PCA is a common multi-variable statistical method and one of the most widely used data dimensionality reduction algorithms. The essence of PCA is to project data samples in high-dimensional space into low-dimensional space via the K-L transform while preserving the original data features as much as possible. The PCA mainly achieves the dimensionality reduction of the original matrix by retaining components with significant variance and a large amount of information and removing components with a small difference and insufficient information [40,41].

Assume X is the input dataset $\left(X=\left[x_{1},\;x_{2},\ldots .,x_{n}\right]\right)$ of P dimensional, it is transformed into a smaller dimension set Y of L dimension (L < P), the Y represent the principal component of X, the process is as follow: (a) calculate the mean, variance and covariance of the spectral data; (b) calculate the eigenvalues and eigenvectors; (c) the eigenvectors are ordered according to the eigenvalues from highest to lowest. The components whose cumulative variance contribution value is greater than 90% are selected as spectral feature data to represent the original spectra.

### 3.6. Probabilistic Neural Networks (PNNs)

PNNs is a forward-propagating neural network that often learns more quickly than many back-propagation networks neural models. It is because PNNs combine Bayesian decision theory and density function estimation to determine the type of samples. The network structure of PNNs consists of four layers: the input layer, a hidden (pattern) layer, a summation layer, and an output layer [42,43,44,45].

### 3.7. Genic Algorithm Optimization SVM Parameters Algorithm (GA-SVM)

#### 3.7.1. The Support Vector Machine Algorithm 

The support vector machine (SVM) has been utilized as one of the most popular schemes for the classification of data in the past decades [46,47]. It is a margin-based classifier with excellent generalization features. In the classification problem, the SVM transforms the input space into a high-dimensional space using a nonlinear transformation defined by an inner product function. In this space, the SVM can find an optimal hyper-plane between different class data [48,49]. The data are not linearly separable in low dimensions, and it is easier to search for a separating hyper-plane when mapped into high-dimensional space. 

The parameters in the SVM algorithm, such as the penalty coefficient C and the Gaussian kernel δ, have a significant impact on the classification results. Therefore, appropriate C and δ should be selected to make the model achieve the best effect.

#### 3.7.2. The Flowchart of the GA-SVM Algorithm 

**Figure 5:**
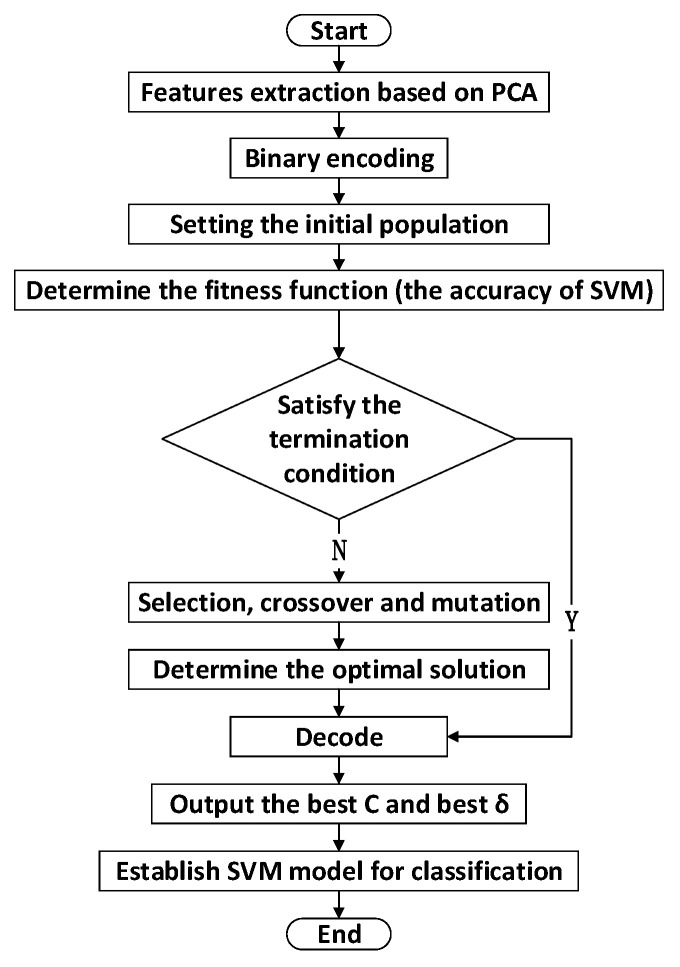
GA-SVM modeling flow chart.

The Genetic Algorithm (GA) is an adaptive heuristic search algorithm for population evolution through continuous and efficient selection, crossover, mutation, and other operations [50,51]. In this work, the GA is used to optimize the penalty parameter C and the kernel parameter δ of SVM and the GA-SVM prediction model based on PCA is established. In the GA-SVM algorithm, the correct classification rate of the SVM is used as the fitness function of the GA. The algorithm flow is shown in Figure 5, which includes the following steps:

Step 1: Feature spectra extraction. According to the mean value and standard deviation of the original data, the data set is standardized to a unit scale, and PCA is used to extract spectral features.

Step 2: Binary encoding and setting the initial population. Determine the parameters of initial population, including the size of the population, the number of iterations, crossover rate, and mutation rate. Additionally, the binary encoding method is used to determine the penalty function C and function parameter δ.

Step 3: Determine fitness function. The corresponding parameter C and δ are decoded to obtain the actual parameter optimization solutions. The obtained SVM parameters C and δ are used for learning and training with the LIBSVM model, and the classification accuracy is obtained by testing the test data in the well-trained model, which will be used to calculate the fitness function.

Step 4: Termination criteria. If the termination criteria of the genetic algorithm are satisfied, the process ends and the optimal parameters are selected and input to the SVM model. Otherwise, perform operations (selection, crossover and mutation) to generate a new generation of population, and finally determine the optimal solution.

## 4. Conclusions

In the present work, the feasibility of identifying oil spill species based on the concentration-emission matrix (CEM), under the condition of weathering and different water environment disturbance conditions, is studied. PCA can be used to extract the spectra feature, combined with the two pattern recognition methods to achieve rapid identification of different oil samples. The result show that GA-SVM has the highest classification accuracy for six kinds of oils, which can reach 100%. The average classification accuracy is 20% higher than the PNNs. 

This work demonstrates that the CEM spectra overcome the problem that traditional fluorescence spectroscopy cannot effectively reflect the changes in spectral characteristics caused by concentration. Additionally, CEM spectra combined with PCA and GA-SVM can realize the accurate identification of different kinds of oils. The works promise to establish a portable oil source discrimination technique, which can be given to help achieve rapid and cost-effective oil spill source recognition.

## Figures and Tables

**Table 1:** Parameters for the Genic Algorithm optimization Support Vector Machine (GA-SVM) and Probabilistic Neural Networks (PNNs).

GA-SVM		PNNs	
the size of the population	20	the spread of radial basis functions	0.1
the maximum iteration number	200		
the mutation rate	0.9		
the penalty coefficient C	[0,100]		
the Gaussian kernel δ	[0,100]		

**Table 2:** Test set classification results by PNNs and GA-SVM.

Name	Target Label	Classification Accuracy (%) [Right Samples/Samples]	
		PNNs	GA-SVM
Crude oil	1	100% [10/10]	100% [10/10]
0#diesel	2	90% [9/10]	100% [10/10]
Heavy oil	3	20% [2/10]	100% [10/10]
Motor oil 20w-40	4	100% [10/10]	100% [10/10]
92#gasoline	5	100% [10/10]	100% [10/10]
Shell helix 10w-40	6	70% [7/10]	100% [10/10]
Average accuracy		80% [48/60]	100% [60/60]

## References

[1] Profumo A., Gorroni A., Guarnieri S.A., Mellerio G.G., Cucca L., Merli D. (2020). GC-MS qualitative analysis of the volatile, semivolatile and volatilizable fractions of soil evidence for forensic application: A chemical fingerprinting. Talanta.

[2] Sales C., Portolés T., Johnsen L., Danielsen M., Beltran J. (2019). Olive oil quality classification and measurement of its organoleptic attributes by untargeted GC–MS and multivariate statistical-based approach. Food Chem..

[3] De Oteyza T.G., Grimalt J.O. (2006). GC and GC–MS characterization of crude oil transformation in sediments and microbial mat samples after the 1991 oil spill in the Saudi Arabian Gulf coast. Environ. Pollut..

[4] Xiao Y., Yan B., Zhang X., Chang X., Li M. (2020). Study the diffusion characteristics of rejuvenator oil in aged asphalt binder by image thresholding and GC–MS tracer analysis. Constr. Build. Mater..

[5] Wang Z., Li K., Fingas M., Sigouin L., Ménard L. (2002). Characterization and source identification of hydrocarbons in water samples using multiple analytical techniques. J. Chromatogr. A.

[6] Danish M., Nizami M. (2019). Complete fatty acid analysis data of flaxseed oil using GC-FID method. Data Brief.

[7] Ayyldz M.F., Fndkolu M.S. (2020). A simple and efficient preconcentration method based on vortex assisted reduced graphene oxide magnetic nanoparticles for the sensitive determination of endocrine disrupting compounds in different water and baby food samples by GC-FID. J. Food Compos. Anal..

[8] Da Silva S.A., Sampaio G.R., Torres E.A.F.D.S. (2017). Optimization and validation of a method using UHPLC-fluorescence for the analysis of polycyclic aromatic hydrocarbons in cold-pressed vegetable oils. Food Chem..

[9] Ariffin A.A., Ghazali H., Kavousi P. (2014). Validation of a HPLC method for determination of hydroxymethylfurfural in crude palm oil. Food Chem..

[10] Obisesan K.A., Jiménez-Carvelo A.M., Cuadros-Rodríguez L., Ruisánchez I., Callao M.P. (2017). HPLC-UV and HPLC-CAD chromatographic data fusion for the authentication of the geographical origin of palm oil. Talanta.

[11] Shi X., Ma J., Zheng R., Wang C., Kronfeldt H.-D. (2012). An improved self-assembly gold colloid film as surface-enhanced Raman substrate for detection of trace-level polycyclic aromatic hydrocarbons in aqueous solution. J. Raman Spectrosc..

[12] Shi X., Kwon Y.-H., Ma J., Zheng R., Wang C., Kronfeldt H.-D. (2012). Trace analysis of polycyclic aromatic hydrocarbons using calixarene layered gold colloid film as substrates for surface-enhanced Raman scattering. J. Raman Spectrosc..

[13] Wang C., Chen B., Zhang B., He S., Zhao M. (2013). Fingerprint and weathering characteristics of crude oils after Dalian oil spill, China. Mar. Pollut. Bull..

[14] Xiao F., Liu L., Zhang Z., Wu K., Xu Z., Zhou C. (2014). Conflicting sterane and aromatic maturity parameters in Neogene light oils, eastern Chepaizi High, Junggar Basin, NW China. Org. Geochem..

[15] Zhao P., Rui H. (2021). A simple and quick method to detect adulterated sesame oil using 3D fluorescence spectra. Spectrochim. Acta A Mol. Biomol. Spectrosc..

[16] Divya O., Mishra A.K. (2008). Chemometric Study of Excitation—Emission Matrix Fluorescence Data: Quantitative Analysis of Petrol—Kerosene Mixtures. Appl. Spectrosc..

[17] Palombi L., Alderighi D., Cecchi G., Raimondi V., Toci G., Lognoli D. (2013). A fluorescence LIDAR sensor for hyper-spectral time-resolved remote sensing and mapping. Opt. Express.

[18] Rayner D.M., Szabo A.G. (1978). Time-resolved laser fluorosensors: A laboratory study of their potential in the remote characterization of oil. Appl. Opt..

[19] Insausti M., Romano C., Pistonesi M.F., Band B.S.F. (2013). Simultaneous determination of quality parameters in biodiesel/diesel blends using synchronous fluorescence and multivariate analysis. Microchem. J..

[20] Bugden J., Yeung C., Kepkay P., Lee K. (2008). Application of ultraviolet fluorometry and excitation–emission matrix spectroscopy (EEMS) to fingerprint oil and chemically dispersed oil in seawater. Mar. Pollut. Bull..

[21] Mirnaghi F.S., Soucy N., Hollebone B.P., Brown C.E. (2018). Rapid fingerprinting of spilled petroleum products using fluorescence spectroscopy coupled with parallel factor and principal component analysis. Chemosphere.

[22] Panigrahi S.K., Mishra A.K. (2020). Inner filter effect mediated red-shift in synchronous and total synchronous fluorescence spectra as a tool to monitor quality of oils and petrochemicals. Fuel.

[23] Patra D. (2001). Concentration dependent red shift: Qualitative and quantitative investigation of motor oils by synchronous fluorescence scan. Talanta.

[24] Patra D. (2008). Distinguishing motor oils at higher concentration range by evaluating total fluorescence quantum yield as a novel sensing tool. Sens. Actuators B Chem..

[25] Zhou Z., Liu Z., Guo L. (2013). Chemical evolution of Macondo crude oil during laboratory degradation as characterized by fluorescence EEMs and hydrocarbon composition. Mar. Pollut. Bull..

[26] Zhou Z., Guo L., Shiller A.M., Lohrenz S.E., Asper V.L., Osburn C.L. (2013). Characterization of oil components from the Deepwater Horizon oil spill in the Gulf of Mexico using fluorescence EEM and PARAFAC techniques. Mar. Chem..

[27] Wang C.-Y., Li W.-D., Luan X.-N., Zhang D.-Y., Zhang J.-L., Zheng R. (2010). [Fingerprint discrimination technique of spill oil based on concentration auxiliary parameter fluorescence spectra]. Guang Pu Xue Yu Guang Pu Fen Xi.

[28] Wang C., Shi X., Li W., Wang L., Zhang J., Yang C., Wang Z. (2016). Oil species identification technique developed by Gabor wavelet analysis and support vector machine based on concentration-synchronous-matrix-fluorescence spectroscopy. Mar. Pollut. Bull..

[29] Phaneendra K.B.L.N. (2020). A new framework for hyperspectral image classification using Gabor embedded patch based convolution neural network. Infrared Phys. Technol..

[30] Jaouher B., Lotfi S. (2015). Linear feature selection and classification using PNN and SFAM neural networks for a nearly online diagnosis of bearing naturally progressing degradations. Eng. Appl. Artif. Intell..

[31] Zhou X., Jiang W., Tian Y., Shi Y. (2010). Kernel subclass convex hull sample selection method for SVM on face recognition. Neurocomputing.

[32] Li Y., Tian X., Song M., Tao D. (2015). Multi-task proximal support vector machine. Pattern Recognit..

[33] Wang X.-Y., Zhang B.-B., Yang H.-Y. (2013). Active SVM-based relevance feedback using multiple classifiers ensemble and features reweighting. Eng. Appl. Artif. Intell..

[34] Xu K.-K., Li H.-X., Yang H.-D. (2017). Dual least squares support vector machines based spatiotemporal modeling for nonlinear distributed thermal processes. J. Process. Control..

[35] Gangsar P., Tiwari R. (2017). Comparative investigation of vibration and current monitoring for prediction of mechanical and electrical faults in induction motor based on multiclass-support vector machine algorithms. Mech. Syst. Signal Process..

[36] Ríos-Reina R., Elcoroaristizabal S., Ocaña-González J.A., García-González D., Amigo J.M., Callejón R.M. (2017). Characterization and authentication of Spanish PDO wine vinegars using multidimensional fluorescence and chemometrics. Food Chem..

[37] Chang B.M., Tsai H.H., Yen C.Y. (2016). SVM-PSO based rotation-invariant image texture classification in SVD and DWT domains. Eng. Appl. Artif. Intell..

[38] Zhang X., Chen W., Wang B., Chen X. (2015). Intelligent fault diagnosis of rotating machinery using support vector machine with ant colony algorithm for synchronous feature selection and parameter optimization. Neurocomputing.

[39] Li K., Wang L., Wu J., Zhang Q., Liao G., Su L. (2018). Using GA-SVM for defect inspection of flip chips based on vibration signals. Microelectron. Reliab..

[40] Gallo C., Capozzi V., Crescenzio G., Vito C. (2019). Feature Selection with Non Linear PCA: A Neural Network Approach. J. Appl. Math. Phys..

[41] Ueda T., Hoshiai Y. (1997). Application of principal component analysis for parsimonious summarization of dea inputs and/or outputs. J. Oper. Res. Soc. Japan.

[42] Altınel B., Ganiz M.C., Diri B. (2015). A corpus-based semantic kernel for text classification by using meaning values of terms. Eng. Appl. Artif. Intell..

[43] Chen Z., Huang A., Qiang X. (2020). Improved neural networks based on genetic algorithm for pulse recognition. Comput. Biol. Chem..

[44] Zhang Y., Dong Z.-C., Wang S., Ji G., Yang J. (2015). Preclinical Diagnosis of Magnetic Resonance (MR) Brain Images via Discrete Wavelet Packet Transform with Tsallis Entropy and Generalized Eigenvalue Proximal Support Vector Machine (GEPSVM). Entropy.

[45] Wan C., Cao W., Cheng C. (2014). Research of Recognition Method of Discrete Wavelet Feature Extraction and PNN Classification of Rats FT-IR Pancreatic Cancer Data. J. Anal. Methods Chem..

[46] Xu M., Su H., Li Y., Li X., Liao J., Niu J., Lv P., Zhou B. (2019). Stylized Aesthetic QR Code. IEEE Trans. Multimed..

[47] Bi K., Qiu T. (2019). An intelligent SVM modeling process for crude oil properties prediction based on a hybrid GA-PSO method. Chin. J. Chem. Eng..

[48] Zhang D., Han J., Zhao L., Meng D. (2018). Leveraging Prior-Knowledge for Weakly Supervised Object Detection Under a Collaborative Self-Paced Curriculum Learning Framework. Int. J. Comput. Vis..

[49] Abe S. (2015). Fuzzy support vector machines for multilabel classification. Pattern Recognit..

[50] Wang L., Xu G., Wang J., Yang S., Guo L., Yan W. GA-SVM based feature selection and parameters optimization for BCI research. Proceedings of the 2011 Seventh International Conference on Natural Computation.

[51] Wang S.-T., Yuan Y.-Y., Zhu C.-Y., Kong D., Wang Y.-T. (2019). Discrimination of polycyclic aromatic hydrocarbons based on fluorescence spectrometry coupled with CS-SVM. Measurement.

